# Biohybrid Micro- and Nanorobots for Intelligent Drug Delivery

**DOI:** 10.34133/2022/9824057

**Published:** 2022-02-10

**Authors:** Jinhua Li, Lukas Dekanovsky, Bahareh Khezri, Bing Wu, Huaijuan Zhou, Zdenek Sofer

**Affiliations:** ^1^School of Medical Technology, Beijing Institute of Technology, Beijing 100081, China; ^2^Department of Inorganic Chemistry, University of Chemistry and Technology Prague, Technicka 5, 166 28 Prague 6, Czech Republic

## Abstract

Biohybrid micro- and nanorobots are integrated tiny machines from biological components and artificial components. They can possess the advantages of onboard actuation, sensing, control, and implementation of multiple medical tasks such as targeted drug delivery, single-cell manipulation, and cell microsurgery. This review paper is to give an overview of biohybrid micro- and nanorobots for smart drug delivery applications. First, a wide range of biohybrid micro- and nanorobots comprising different biological components are reviewed in detail. Subsequently, the applications of biohybrid micro- and nanorobots for active drug delivery are introduced to demonstrate how such biohybrid micro- and nanorobots are being exploited in the field of medicine and healthcare. Lastly, key challenges to be overcome are discussed to pave the way for the clinical translation and application of the biohybrid micro- and nanorobots.

## 1. Introduction

Microrobotics is dedicated to the research and development of artificial machines with the maximum size on the micron scale for a wide range of real-world applications. This emerging research field has received ever-increasing attention, especially after molecular machines were selected as the topic of the Nobel Prize in Chemistry 2016. In the talk “There's Plenty of Room at the Bottom,” Richard P. Feynman envisioned the new field of small-scale machines [1]. From the idea “swallow the surgeon” to the later movie “Fantastic Voyage,” these micro- and nanorobots are expected to hold great promise for a variety of biomedical applications, typically targeted drug delivery, minimally invasive surgery, and single-cell manipulation [2–7].

The purpose of the medical microrobotics is to develop and deploy large numbers of micro/nanomachines (capable of physical, chemical, or biological propulsion, programmability, and reconfigurability) to carry out diverse medical tasks (e.g., delivering drugs in situ, generating local hyperthermia, targeting diseased cells, and performing cell microsurgery) inside the complex body conditions. Nevertheless, existing challenges in materials design, mass production, biocompatibility, and control over locomotion and functionality need further efforts to overcome, thereby releasing the translational potential of medical microrobots for the clinic [4, 8]. Conventional fabrication techniques of micro/nanorobots encompass the electroless plating [9], template-assisted electrodeposition [10], physical vapor deposition [11], strain engineering [12], 3D printing [13, 14], capillary micromolding [15], material assembly [16], bioinspired design [17–19], and biohybridizing method [20].

**Scheme 1:**
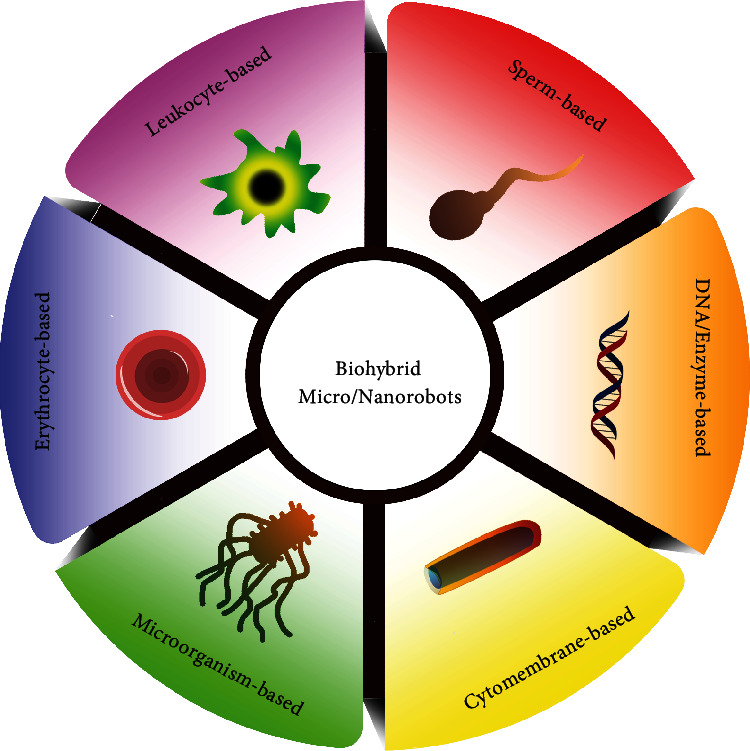
Summary of various biohybrid micro- and nanorobots.

Since not all the biohybrid micro/nanosystems fall to micro/nanorobots, it is necessary to clarify the definition of biohybrid micro- and nanorobots. The biohybrid micro- and nanorobots refer to functional micro- and nanorobots that comprise biological components (e.g., DNA, enzyme, cytomembrane, and cells) and artificial components (e.g., inorganic or polymer particles). They can inherit the parental biological properties, onboard actuation, and sensing capabilities [21]. In recent years, great efforts have been made by researchers to this emerging field of biohybrid micro/nanorobots and several reviews have been published as valuable reference resources on relevant specific topics [20, 22–25]. In this review, we will first highlight different types of biohybrid micro- and nanorobots concisely, as summarized in Scheme 1. Afterward, we will introduce the representative medical applications of biohybrid micro- and nanorobots as intelligent drug delivery systems. Finally, an outlook on the future directions of biohybrid micro- and nanorobots will be discussed.

## 2. Biohybrid Micro/Nanorobots

### 2.1. DNA-, Enzyme-, or Cytomembrane-Based Nanorobots

**Figure 1:**
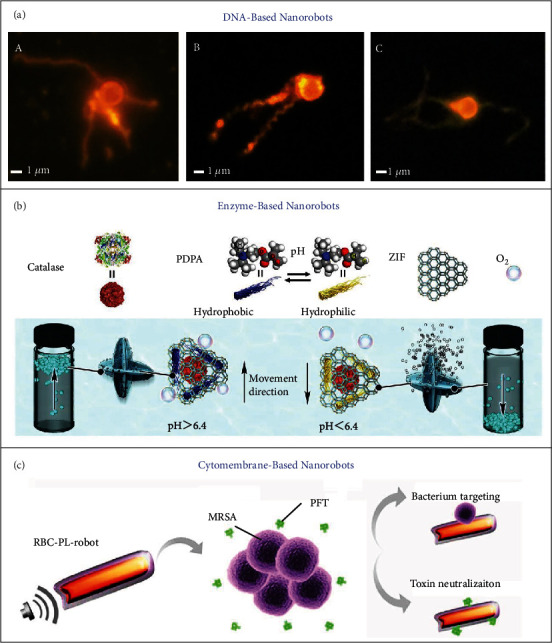
(a) Magnetic microswimmers hybridized with DNA flagellar bundles of straight 8HT (A), twisted 8HT (B), and supertwisted 13HT (C). Reproduced with permission from Reference [28]. Copyright 2016, American Chemical Society. (b) Locomotion mechanism of catalase-propelled submarine-like MOF micromotors. Reproduced with permission from Reference [36]. Copyright 2019, Elsevier Ltd. (c) Hybrid RBC-PL-robots for performing medical tasks. Reproduced with permission from Reference [44]. Copyright 2018, The Authors, exclusive licensee American Association for the Advancement of Science.

The interactions with extraordinary specificity between complementary oligonucleotides in a double helix enable DNA a useful building material and the structures of branch junctions between DNA double helices make it possible to create complicated 3D objects through self-assembly [26, 27]. Maier and coworkers reported the development of magnetic microswimmers with the DNA-based flagellar bundles, as shown in Figure 1(a) [28]. The DNA flagella were attached to magnetic iron oxide microparticles (1 *μ*m) through hybridization of complementary DNA strands, thereby producing the biohybrid magnetic microrobots driven by the homogeneous magnetic field rotating perpendicular to swimming direction. DNA nanorobots have shown great potential for tumor-targeted drug delivery and vaccination for precision cancer (immuno) therapy [29–31]. Nevertheless, their limited stability in the physiological environment may cause insufficient circulation and biodistribution, which requires more efforts to enhance their resistance against damage.

Enzymes are responsible for boosting a variety of metabolic activities in the living systems [32, 33]. The enzymatic catalysis involves the transformation of the substrate (reactant) into product and is accompanied by the release of energy. The mechanical forces produced in these enzymatic reactions are competent to trigger the enzymatic propulsion in a directional way in response to the substrate gradients (i.e., chemotaxis) [34, 35]. As a consequence, immobilizing enzymes on the surface of a particle or sticking enzymes on a solid support can lead to self-propelled carriers or fluid pumps with numerous promising applications. Self-propelled submarine-like micromotors were created on the basis of metal-organic frameworks (MOFs) that encapsulate catalase as the engine and poly(2-diisopropylamino)ethyl methacrylate (PDPA) as the pH-responsive, hydrophobic/hydrophilic phase-shifting component, and could result in the ascending and descending vertical motion controlled by buoyancy, as shown in Figure 1(b) [36]. Somasundar and coworkers demonstrated both positive and negative chemotaxis on the catalase- and urease-coated liposome motors (liposomal protocells) [37]. In a recent study by Hortelao and coworkers [38], the swarming behaviors of the urease-powered nanomotors were well tracked, monitored, and analyzed by using the positron emission tomography (PET) technique. Active swarming dynamics and real-time imaging tracking are expected to make an important step forward in the area of biomedical nanorobotics and pave the way towards their theranostic applications.

As exogenous invaders, synthetic micro/nanocarriers for in vivo drug delivery can easily trigger passive immune clearance, increase retention effect due to bioadhesion and reticuloendothelial system, and finally cause low therapeutic efficacy. To solve these issues, recently, a cell membrane cloaking approach has been developed as a novel surface engineering strategy from the perspective of biology and immunology, proving powerful for promoting the performances of synthetic micro/nanocarriers in vivo [39, 40]. Cell membrane-camouflaged micro/nanomotors are able to not only transform surrounding energy into directional, autonomous locomotion but also inherit the natural functions of cell membranes, with the guidable property by physical fields (magnetic field, ultrasound, light, etc.) and chemical fuel/chemoattractant [41]. Wu and coworkers developed ultrasonic nanomotors by fusing biocompatible Au nanowire motors and red blood cell (RBC) nanovesicles [42] and later created magnetic helical Ni/Au/Pd nanorobots cloaked with the plasma membranes of human platelets (PLs) [43]. These biohybrid nanorobots could exhibit efficient propulsion within the whole blood over a long period of time. They further achieved the construction of ultrasonic Au nanowire robots camouflaged with hybrid RBC and PL membranes (Figure 1(c)) [44]. Such biohybrid nanorobots demonstrated fast, efficient, and prolonged ultrasonic propulsion in the whole blood, without significant biofouling. Collectively, the produced micro/nanorobots are able to acquire sophisticated structures and functionalities through the biohybridizing approach, thereby holding promise for implementing complex medical tasks that cannot be done solely by artificial active particles.

### 2.2. Leukocyte-Based Hybrid Microrobots

**Figure 2:**
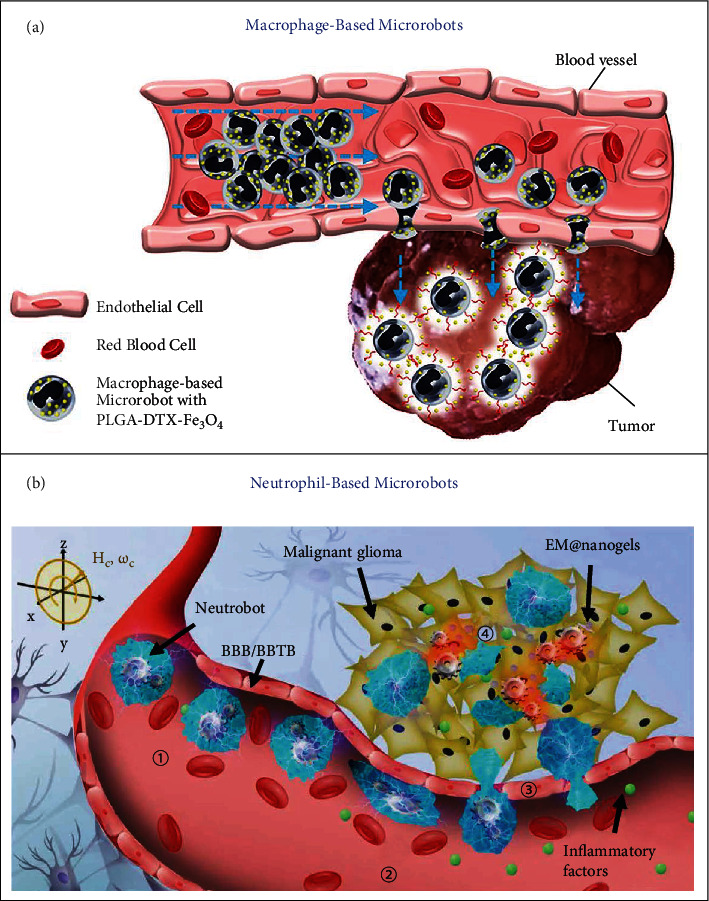
(a) Macrophage-based biohybrid microrobots for active tumor therapy. Reproduced with permission from Reference [50]. Copyright 2016, The Authors, licensed under a Creative Commons Attribution 4.0 International License. (b) Active drug delivery of dual-responsive neutrobots towards the malignant glioma. ① Active cumulation of neutrobots towards the glioma under an external magnetic field. ② Chemotaxis of neutrobots along the gradient of the inflammatory factors. ③ BBB penetration of neutrobots. ④ Local release of PTX from neutrobots inside the malignant glioma. Note: BBB/BBTB = blood-brain barrier/blood-brain tumor barrier. Reproduced with permission from Reference [56]. Copyright 2021, The Authors, exclusive licensee American Association for the Advancement of Science.

Leukocytes, also referred to as white blood cells (WBCs), are the cells of the body's immune system and participate in the protection of the body against neoplastic/infectious diseases and foreign invaders [45]. Considering their intrinsic properties/functions such as chemotaxis and secretion activity, leukocytes have been engineered into biohybrid microrobots. Macrophages are an essential part of the innate immune system and play an important role in development, homeostasis, diseases, and other physiological activities [46]. Macrophages are derived from monocytes [47] and their phenotypes and functions can be modulated by tailoring environmental cues [48]. Using mouse J774A-1 macrophages, Yasa and coworkers demonstrated the macrophage-based biohybrid microrobots (so-called “immunobots”), which were able to combine the immunomodulatory capacity of macrophages and the navigable mobility of 3D-printed microswimmers for targeted immunotherapeutics [49]. Previously, on the basis of macrophage recruitment/homing in tumors, researchers developed macrophage-based microrobots as vehicles to deliver anticancer drugs to the tumor sites (Figure 2(a)) [50]. Recently, dual-targeting macrophage-based microrobots were developed with controllability by inherent chemotaxis and external magnetic field to implement NIR-responsive precision drug release at tumor regions in a spatiotemporally controlled pattern [51]. In addition, monocyte-based microrobots have been created with chemotactic transmigrating motility similar to actual monocytes [52]. Neutrophils, also known as polymorphonuclear neutrophils (PMNs), are the most abundant granulocyte type and occupy 40%~70% of leukocytes in the human body, serving as an essential component of the innate immune system [53, 54]. Neutrophils with native chemotaxis have been converted into self-guided biohybrid micromotors through phagocytosing mesoporous silica nanoparticles (MSNs) for high drug-loading capacity [55]. Neutrophil-based microrobots (“Neutrobots”) were capable of the active delivery of cargos into the malignant glioma in vivo (Figure 2(b)) [56]. The unique advantage of immunobots lies in that they can escape the phagocytosis and removal by the mononuclear phagocyte system (MPS) and exhibit chemotactic locomotion toward the diseased sites (such as infection, tumor, or inflammation). Therefore, immunocyte-based microrobots have the capability to autonomously target diseased tissues, actively deliver therapeutic drugs, and locally release the drugs.

### 2.3. Erythrocyte- and Spermatozoa-Based Microrobots

**Figure 3:**
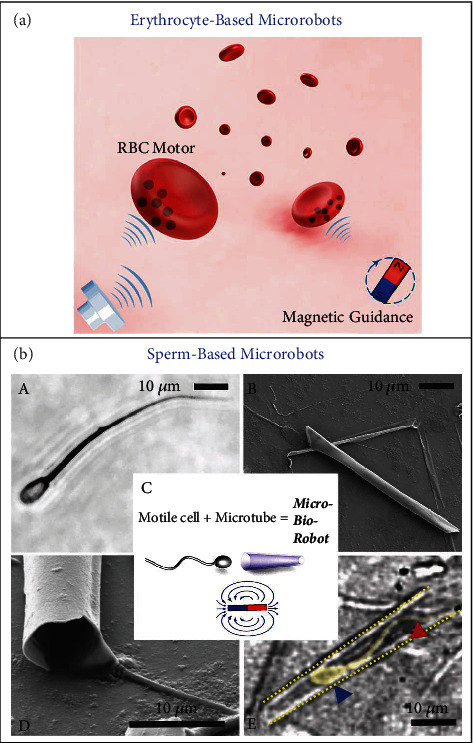
(a) Schematic illustration of magnetically navigated, ultrasonically propelled RBC micromotors in the whole blood. Reproduced with permission from Reference [58]. Copyright 2014, American Chemical Society. (b) Sperm flagella-driven micro-bio-robots. (A) Optical image of a bull spermatozoon. (B, D) SEM images of rolled-up Ti/Fe microtube on glass with a sperm at the opening of tube. (C) Illustrative fabrication of a micro-bio-robot through trapping a motile sperm inside a Ti/Fe microtube for magnetic remote control. (E) Optical image of a sperm (yellow shadow) trapped inside a Ti/Fe microtube (yellow dots). Note: blue arrow = sperm head; red arrow = sperm flagellum. Reproduced with permission from Reference [64]. Copyright 2013, WILEY-VCH Verlag GmbH & Co. KGaA, Weinheim.

Erythrocytes, also referred to as red blood cells (RBCs), have been serving as an attractive endogenous cargo-carrier material for drug delivery over the past decades, and researchers have achieved numerous advancements in developing erythrocyte-based carriers for drug delivery [57]. Magnetic iron oxide NPs (20 nm) have been incorporated to transform native-mouse RBCs into functional micromotors capable of ultrasonic propulsion, magnetic guidance, and preservation of the structural and biological features of regular erythrocytes (Figure 3(a)) [58]. In addition to their excellent biocompatibility, RBCs are the most abundant cell in the human body and possess long circulation half-life (~120 days in human blood), which are beneficial for establishing erythrocyte microrobots to target diseased sites and deliver drug molecules. Besides, platelets have been also exploited as a promising cargo-carrier material for targeted drug delivery [59]. Recently, endogenous platelet-based enzyme-powered Janus micromotors have been developed through the asymmetric immobilization of urease onto the partial surface of native platelets [60]. Platelets have native selectivity to injured tissues and tumor microenvironment. Together with their longer circulation time (8~10 days), platelet-based microrobots have the potential for local accumulation and drug delivery within a targeted tissue.

Sperms are the male reproductive cells, and mammals generate motile sperms (spermatozoa), which have a tail called flagellum and exhibit chemotaxis that is important for fertilization [61]. Motile sperms have been converted into robotic microswimmers (so-called “spermbots”), in which the sperms act as the active component [62, 63]. Magdanz and coworkers demonstrated the first example of developing a sperm-based hybrid micro-bio-robot that can be driven by sperm flagella, as shown in Figure 3(b) [64]. A single motile sperm cell was able to enter a magnetic Ti/Fe microtube (50 *μ*m long), being trapped inside the tube. Such a micro-bio-robot could be magnetically navigated to a predefined site. The decrease of microtube length to 20 *μ*m and the addition of caffeine lead to the performance improvement of such spermbots [65]. Spermbot-based drug delivery systems can take advantage of the rheotaxis and thigmotaxis of sperms to reach a targeted site and release drugs locally.

### 2.4. Microorganism-Based Hybrid Microrobots

**Figure 4:**
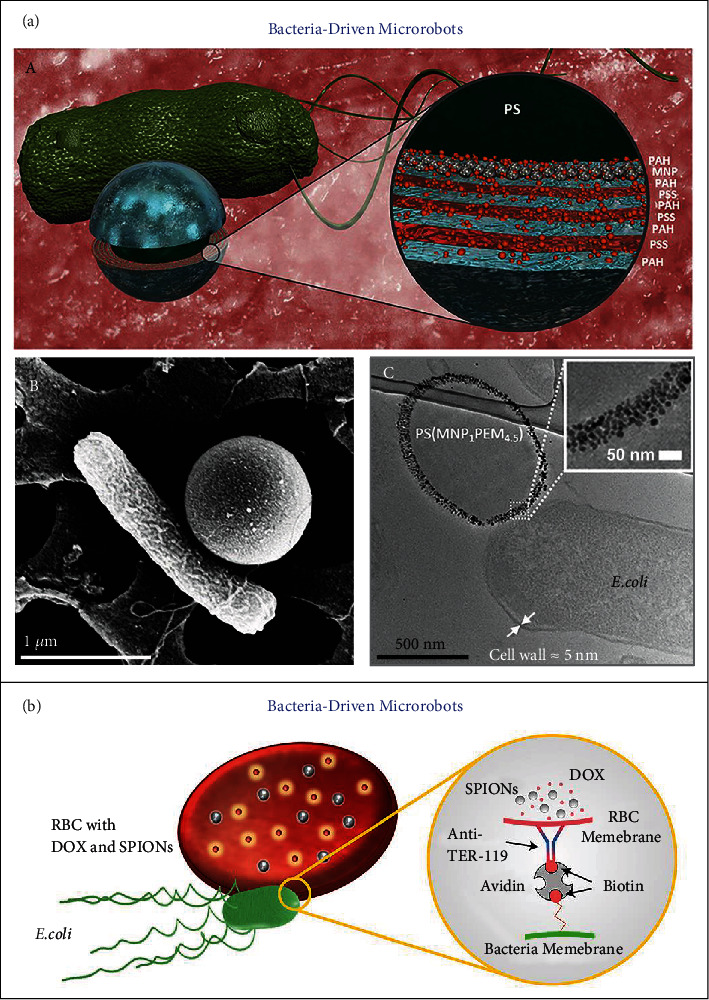
(a) Bacteria-driven microswimmers on the basis of PEM-MNP microparticles attached to E. coli MG1655 bacteria. (A) Schematic design of bacteria-driven microswimmers for active targeted drug delivery. Note: PS = polystyrene microparticle (1 *μ*m diameter). (B) SEM image of one single PS(MNP_1_PAH/PSS)_4_PAH-attached bacterium. (C) TEM image of thin section of a microswimmer. Inset: TEM image of monolayer of MNPs. Reproduced with permission from Reference [71]. Copyright 2017, American Chemical Society. (b) Magnetically guided, bacterially driven RBC microswimmers for active drug delivery. Reproduced with permission from Reference [72]. Copyright 2018, The Authors, exclusive licensee American Association for the Advancement of Science.

Bacteria, one of the major groups of microorganisms, can participate in the development of human health and diseases in a close and dynamic manner. Bacteria have been exploited as promising delivery systems for diverse biomedical purposes [66]. Typically, with the integration of bioengineering and biohybrid strategies, bacteria-based microrobots have been widely developed for targeted drug delivery systems [22, 67]. Mostaghaci and coworkers developed the biohybrid microswimmers driven by the motile E. coli MG1655 bacteria (so-called “bacteriabots”) for bioadhesion to epithelial cells and for targeted drug delivery toward the epithelial cells in urinary or gastrointestinal tracts [68]. Owing to the intrinsic chemotaxis of bacteria [69], these bacteriabots have the capacity to exhibit collective chemotactic behavior [70]. They further established bacteria-driven microswimmers loaded with anticancer drug DOX and magnetic Fe_3_O_4_ nanoparticles (Figure 4(a)) [71]. Such microswimmers could exhibit the biased (chemotactic guiding) and directional (magnetic steering) locomotion for being navigated and targeted to the specific cells. Being driven by the motile E. coli MG1655 bacteria, soft RBC-based microswimmers were developed as autologous carriers for active and guided DOX delivery, as illustrated in Figure 4(b) [72]. Coupled with bacteria-enabled onboard propulsion, the loaded SPIONs could empower the external magnetic navigation of RBC microswimmers that preserved the deformability and attaching stability of natural RBCs. In addition to bacteria, fungi [73, 74] and microalgae [75–78] components have been incorporated into the design of biohybrid microrobots. Integrated with various microorganisms, the hybrid microrobots have the potential to make use of the taxis behaviors of microorganisms in response to diverse environmental factors such as light, oxygen, heat, and magnetic field. Moreover, bioengineered microorganisms are able to produce therapeutic substances and even modulate immune microenvironment, which are expected to increase the functionalities of the hybrid microrobots for implementing complex medical tasks.

## 3. Drug Delivery Applications

**Figure 5:**
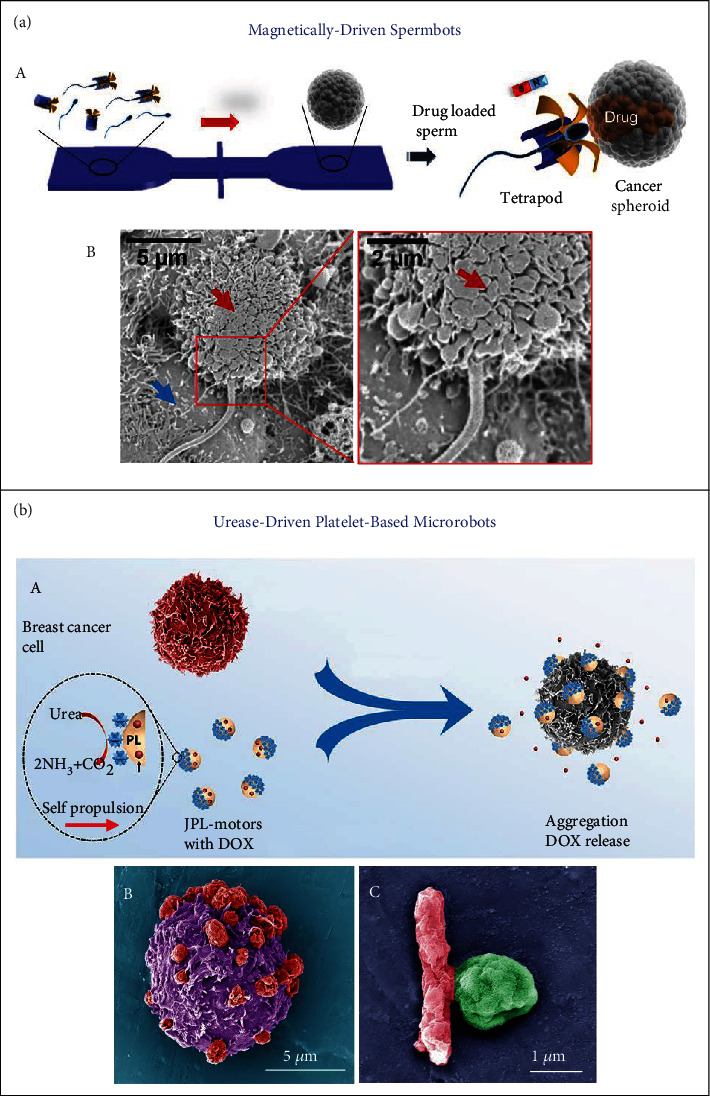
(a) Spermbots for targeted DOX delivery. (A) Illustration of a microfluidic chip for the transport and delivery of drug-loaded sperm. (B) SEM images indicating the fusion of sperm and HeLa cell. Note: red arrows = a cell in apoptosis; blue arrows = live cells. Reproduced with permission from Reference [79]. Copyright 2017, American Chemical Society. (b) Urease-powered Janus platelet robots for enhanced anticancer/antibacterial activity through loading DOX chemodrug or ciprofloxacin (Cip) antibiotic for active, targeted drug delivery. (A) Schematic illustration of DOX-loaded JPL-motors for targeted delivery to MDA-MB-231 breast cancer cells. (B) Pseudocolored SEM image of multiple JPL-motors (red) attaching to a single cancer cell (purple). (C) Pseudocolored SEM image showing the binding between a Cip-loaded JPL-motor (green) and a single E. coli (red). Reproduced with permission from Reference [60]. Copyright 2020, The Authors, exclusive licensee American Association for the Advancement of Science.

Xu and coworkers developed the sperm-driven micromotors as a targeted drug delivery system, revealing promising applications for the treatment of diseases in the female reproductive tract, as shown in Figure 5(a) [79]. Due to the elaborate designs, when such biohybrid spermbots hit tumor walls, they were capable of swimming into the tumor and delivering DOX through the membrane fusion of sperms and cancer cells. The spermbots were also capable of actively swimming against blood flow (rheotaxis) and implementing heparin delivery with the navigation of magnetic field [80]. It was demonstrated that the urease-powered Janus platelet micromotors were able to maintain the intrinsic biofunctionalities of native platelets, thereby enabling the effective targeting of MDA-MB-231 cancer cells and E. coli bacteria for precise release of loaded drugs (Figure 5(b)) [60]. In addition, biohybrid micro- and nanorobots also hold promise for cell-based therapies such as cell microsurgery [81]. The microdagger medibots developed by Srivastava and coworkers were capable of performing single-cell microsurgery and anticancer drug delivery via magnetic control [82]. A cellular drilling action of HeLa cells was demonstrated using the Fe- and Ti-coated biotubes (named as “microdaggers”) under a rotating magnetic field. These microdaggers can stab into the cytomembrane, deliver camptothecin drug into a single cell, and lead to cancer cell death.

Drug-loaded micro- and nanorobots have demonstrated huge potential in vitro. Despite increasing efforts on maintaining their functions in the living body, the complicated physiological environment imposes enormous challenges. Typically, prolonging the circulation time in blood vessels, evading the phagocytosis by phagocytes, and increasing the retention period in targeted sites are needed for largely improving the treatment effect of drug-loaded micro/nanorobots. To this end, biohybrid micro- and nanorobots have been rapidly advancing as long-circulating, biocompatible, and tissue-targeting drug delivery systems in vivo. Due to the integration of biological components, biohybrid micro- and nanorobots are able to exhibit specific sensing ability, taxis behavior, and swarm action, which collectively contribute to improving the drug delivery efficiency, responsivity, and targeting ability. Furthermore, the presence of biological parts can also impart favorable degradability to the biohybrid micro- and nanorobots after accomplishing their tasks in the body.

## 4. Conclusion and Future Outlook

**Figure 6:**
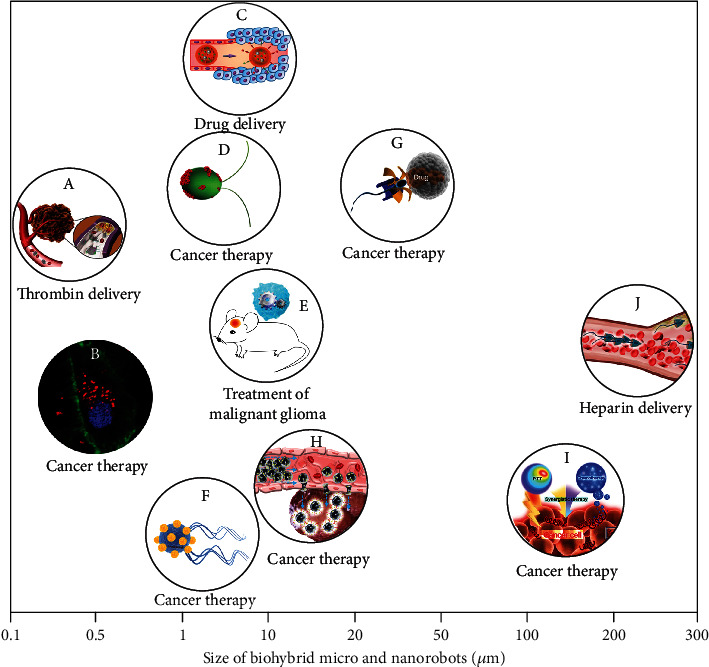
The representative applications of cargo delivery systems versus the size of biohybrid micro- and nanorobots. (a) DNA-based nanorobots for thrombin delivery to tumor-associated blood vessels with the aim to inhibit tumor growth by inducing intravascular thrombosis. Human breast cancer cells (MDA-MB-231) and BALB/c nude mice were used for *in vivo* experiments. Reproduced with permission from Reference [29]. Copyright 2018, Nature Publishing Group. (b) Enzyme-based nanorobots for transport and stimuli-responsive release of drugs ([Ru(bpy)_3_]Cl_2_ or doxorubicin DOX). *In vitro* experiments were conducted by using HeLa cells. Reproduced with permission from Reference [85]. Copyright 2019, American Chemical Society. (c) Erythrocyte-based microrobots for anticancer drug (i.e., DOX) delivery. Reproduced with permission from Reference [86]. Copyright 2020, American Chemical Society. (d) Microalgae (i.e., Chlamydomonas reinhardtii)-based microrobot for anticancer drug (i.e., DOX) delivery. SK-BR-3 breast cancer cells were adopted for *in vitro* experiments. Reproduced with permission from Reference [76]. Copyright 2020, The Authors. (e) Neutrophil-based microrobots for targeted drug delivery in the brain. Under the navigation of a rotating magnetic field, the microrobots can travel across the blood-brain barrier to inhibit the proliferation of tumor cells by releasing the drugs in targeted sites. Reproduced with permission from Reference [56]. Copyright 2016, The Authors. (f) A magnetotactic bacteria-based microrobot with conjugated nanoliposomes, which has the potential to deliver therapy drugs to hard-to-reach regions in solid tumors via the self-propulsion from the flagella and navigation of external magnetic fields. Reproduced with permission from Reference [87] Copyright 2014, American Chemical Society. (g) Sperm-based micromotors with the loading of doxorubicin hydrochloride for active drug delivery. HeLa cell tumor spheroids were used for *in vitro* drug delivery experiments performed in a microfluidic channel. Reproduced with permission from Reference [79]. Copyright 2017, American Chemical Society. (h) Macrophage-based magnetic microrobots loaded with docetaxel for active cancer therapy. The *in vitro* experiments were conducted in a microfluidic channel by using three-dimensional tumor spheroids from 4T1 breast cancer cells or CT26 colorectal carcinoma cells. Reproduced with permission from Reference [50]. Copyright 2016, The Authors. (i) Spirulina-based magnetic helical microrobot loaded with DOX for *in vitro* cancer therapy via controlled pH- and NIR-triggered drug release mode. The 769-P kidney cancer cells and EC109 esophageal cancer cells are used for *in vitro* experiments. Reproduced with permission from Reference [88]. Copyright 2019, American Chemical Society. (j) Sperm-based microrobots for heparin (i.e., a type of anticoagulant agent) transport in flowing blood, which have the potential to treat blood clots or other diseases in the circulatory system. Reproduced with permission from Reference [80]. Copyright 2020, American Chemical Society.

The present review work gives a summary of the recent advancements in rational designs of biohybrid micro- and nanorobots for targeted drug delivery applications, as illustrated in Figure 6. The size of a biohybrid robot is related to the biological template used. For example, using a cell as the template, the robot size is close to the cell size. As emphasized throughout the text, a wide range of biological templates, such as DNA, enzymes, cytomembranes, blood cells (including WBCs, RBCs, and platelets), sperms, and bacteria, has been engineered into biohybrid micro- and nanorobots. Currently, the reported robot sizes are mainly in the range of 1~20 *μ*m. In addition to the already used biological components, such biohybridizing strategies can also apply to other types of microorganisms, mammalian cells [24, 25], or cellular elements for creating functional micro- and nanorobots for specific medical purposes. For example, given the safety concerns over using pathogens, commensal bacteria from the human microbiota are expected to be an emerging paradigm for creating bacteria-based microrobots. The commensal bacteria physiology has close correlation with the host behavior. Therefore, integration of patients' commensal bacteria into designing biohybrid microrobots can promote personalized therapies of human diseases. Photosynthetic microalgae can exploit solar energy to convert CO_2_ and produce pharmaceutical metabolites such as anti-inflammatory, antimicrobial, or antitumor compounds [83]. They have also demonstrated the potential for tissue engineering applications [84]. When transforming microalgae into microrobots, they hold great promise as active, autonomous drug delivery systems. Moreover, current studies have been mainly focused on *in vitro* experiments, but *in vivo* studies are very limited. We herein call on researchers in this field to work together and try more *in vivo* studies on biohybrid micro/nanorobots.

As indicated in Figure 6, cancer therapy is currently the major focus of research on biohybrid micro- and nanorobots for medical applications, especially involving targeted drug delivery and precision tumor killing [23]. Such therapy concept can be rationally extended to treat other diseases. An intelligent and autonomous biohybrid microrobot has the potential to simultaneously sense, search, diagnose, and deliver drugs to cure and care for diseased cells or tissues in the body. The application scenarios of biohybrid micro- and nanorobots also encompass cell microsurgery, gene transfection, cell sorting, assisted fertilization, and in situ tissue engineering. The propulsive forces of biohybrid micro- and nanorobots can result from either the biological components (e.g., the catalysis of enzymes and the motility of microorganisms) or the artificial components (e.g., stimuli-responsive engineered carriers and synthetic attachments). Motion control is crucial for the design and task implementation of biohybrid micro- and nanorobots for various drug delivery applications. Current control methods including magnetic control, optical control, ultrasonic control, electric control, chemical control, and taxis control (e.g., thermotaxis and aerotaxis) can be utilized to manage the locomotion of biohybrid micro- and nanorobots for carrying out specific tasks [20, 25, 89]. The incorporation of physical field-responsive materials can contribute to active, long-range control of the microrobot-based drug delivery systems. Furthermore, the encapsulation of microorganisms or engineered cells into microscaffolds may help them escape from the host immune system and increase their circulation time.

The 3D printing, a versatile manufacturing method of cell microscaffolds, is able to convert the virtual 3D models formed by computer-aided design (CAD) into their corresponding physical 3D constructs through the sequential, layer-by-layer deposition of laser energy (for laser printing), or ink materials (for extrusion printing and inkjet printing) [90–93]. The 3D printing techniques have been widely exploited to develop a variety of functional microrobots and soft robots [13, 94]. On the one hand, biocompatible polymers can be 3D printed into microscaled scaffolds with desired geometries and structures, followed by integrating with living cells or cell-laden hydrogels. On the other hand, cell-laden hydrogels can be directly 3D printed into tissue constructs with predefined sizes and shapes (i.e., 3D bioprinting). Therefore, 3D bioprinting is an emerging technique to engineer multiscale and vascularized muscle tissue constructs from, e.g., myoblasts and cardiomyocytes to power or to actuate such biohybrid soft robots from micrometer to millimeter dimensions or larger scales. In addition, CRISPR-Cas gene editing [95] and synthetic-biology techniques [96] may find wide promising applications in the field of biohybrid micro- and nanorobots through the integration of genetically engineered living cells (e.g., engineered E. coli, yeast cells, microalgae, and macrophages) that act as active biofactory to on-site produce diverse therapeutic compounds for versatile purposes.

Despite the rapid development of biohybrid micro- and nanorobots with ever-increasing functionalities, most of the biohybrid micro- and nanorobots designed for drug delivery purposes are still in their infancy. There is still a long way to go before their commercialization and clinical applications can be achieved. The following major challenges or obstacles should be well considered and addressed for the commercialization. One issue is the lack of a facile, reliable fabrication technique that can achieve the high-throughput manufacturing of biohybrid micro/nanorobots and ensure their homogeneous functionalities. Furthermore, the capacity and application potential have been extensively demonstrated on individual biohybrid micro/nanorobots, while the clinical applications will demand the collective locomotion and coordination of many biohybrid micro- and nanorobots. To this end, the use of physical fields (e.g., magnetic field, light, and ultrasound) to engineer the swarm manipulation and navigation of biohybrid micro/nanorobots may offer a promising solution to their precision operation in the complicated body environment [97]. Moreover, real-time visualization, tracking, and localization of a single biohybrid micro/nanorobot or a swarm of biohybrid micro/nanorobots are crucial for their feedback and external control. For this purpose, the resolution and sensitivity of current imaging techniques (e.g., ultrasound, radiology, fluorescence, photoacoustic tomography, magnetic resonance imaging, and magnetic particle imaging) should be further improved to fulfill the real-time visualization of a single particle or single cell, thereby contributing to the clinical translation and commercialization of biohybrid micro/nanorobots for a wide range of practical applications.

## Data Availability

Data of this paper are available by emailing lijinhua_academia@163.com.
